# Tubercular Ulcers of the Tongue

**Published:** 1892-02-20

**Authors:** 


### SURGERY.

### TUBERCULAR ULCERS OF THE TONGUE.

Until ten years ago little was known or written about tubercular ulcers of the tongue in this country. In 1882 several specimens of the disease were brought forward at the Pathological Society, and Mr. Barker collected and abstracted all the cases published on the Continent up to that date. Since then several cases have been reported, and the disease has found a place in modern text books on surgery.

This form of ulcer is of great clinical interest, for the difficulty of diagnosis from epethelioma is often very considerable. We have lately seen two cases in which the diagnosis of epithelioma was actually made in one case, and was seriously considered in the other. The disease may occur at any age. One of the patients referred to was 55 years of age, a period of life when, of course, epethelioma is common. In tubercular ulcer the glands may be considerably enlarged from tubercular deposits, as in this case, and thus further simulate malignant disease. But there is not so much induration as in cancer. Certainly the tubercular ulcer may be indurated at parts of its edge, as we have seen in these cases; but there is not the indurated base so common in an epithelioma. Their base is generally covered with pale, flabby granulations, and the edge is sharp cut or bevelled. They often begin as little yellow points which ultimately coalesce. Tubercular ulcer is nearly always associated with pulmonary phthisis. It commonly occurs on the tip, or extending from the frenum up to the tip, and occasionally, though rarely, there is more than one ulcer. For many years years no case was recorded in a child, but at a recent meeting of the Pathological Society a case was mentioned by Dr. Pemrose in a child, in which tubercle bacilli were demonstrated. These bacilli have been found in cases brought before the society of late years. At first the tubercular nature of the ulcer was shown only by its microscopic structure of tubercle tissue, except in one case where Mr. Watson Cheyne found bacilli.

These tubercular ulcers, occurring as they do in phthisis, are sometimes found in chest hospitals. We think they are often not diagnosed as tubercular, but supposed to be due to irritation of rough teeth. And so possibly they often are, for the abrasion once formed may allow of bacillary inoculation from the phthisical sputum. It is very suggestive that in Mr. Barker's case, brought before the Pathological Society in 1883, the ulcer followed an abrasion of the tongue from the man holding tin-tacks in his mouth. We do not think that the other theory—that the phthisis is produced by the passage of inspired air over the ulcer—can be accepted, for air would only take up bacilli from a dry surface, not from a moist ulcer; and even with the mouth open during sleep, we cannot conceive of its being dry enough for this. These ulcers are nearly always secondary to the onset of the pulmonary lesion, but in one or two cases seem primary. Tubercular ulcers in their later stage are often very painful. They tend sometimes to heal, and that almost completely, but they invariably break down again, and no case of real cure is recorded.

We have found the tubercular tissue infiltrating the tongue for a depth of half an inch below the ulcer, even when the ulcer was very small. In one case we found large numbers of tubercle bacilli; in another, only three or four in ten sections.

If we are in doubt about the diagnosis, we may under cocaine take a scraping of the ulcer, as Mr. Butlin suggested we might do in doubtful cases of epithelioma, and examine it for tubercle bacilli. We certainly might find them and thus establish the diagnosis; but seeing how few are sometimes present, we must not look on the negative as of any value. We must take great care to cleanse the surface from the bacillary sputum of phthisis which may be present in the mouth, or we may be misled by finding pulmonary bacilli on the ulcer. In cases of difficulty in the diagnosis from epithelioma the absence of cell nests in the scraping will be of help to us in our diagnosis.

What can be done for tubercular ulcers of the tongue? First of all we should be particularly careful not to allow the tongue to be rubbed by rough teeth, or a rough clay pipe, in a phthisical person. In a healthy man it may, perhaps, merely set up an ulcer which will heal on removal of the irritation, unless, indeed, it starts malignant growth; but in a person expectorating bacillary sputum this abrasion may form the point of inoculation, and a tubercular ulcer may develop.

Shall we excise these ulcers? If the ulcer were commonly the primary lesion (as in a few cases it seems to be) we might discuss this question from the point of prevention of secondary lesions just as we might the propriety of castration on this ground; but it is so rarely primary that even in these cases it becomes a question whether there may not be some lurking tubercular pulmonary mischief as yet unrevealed by physical signs. But there is another reason for excision, and that is the pain of these ulcers after they have existed some little time. Looking at the fact that they are so often on the tip, it is evidently not a serious operation to remove them with a fair amount of the surrounding apparently healthy tissue. That this is necessary is shown by the extent to which the surrounding tissues was infiltrated with tubercle in the case already referred to. If we do not excise, then frequent application of cocaine, particularly at the time of taking food, should be made. We doubt if superficial scraping would do much good; it would not get away all the tubercular tissue in many cases, and excision would be a better operation than deep scraping. The ulcer might be removed in a wedge-shaped piece, and the two sides of the v shaped opening brought together and the hæmorrhage from the smaller vessels checked in this way. But it must not be forgotten that in making a wound we are exposing the organ to the risk of further tubercular infection if the disease is secondary to pulmonary phthisis.

